# SPF Rabbits Infected with Rabbit Hepatitis E Virus Isolate Experimentally Showing the Chronicity of Hepatitis

**DOI:** 10.1371/journal.pone.0099861

**Published:** 2014-06-17

**Authors:** Jian Han, Yaxin Lei, Lin Liu, Peng Liu, Junke Xia, Yulin Zhang, Hang Zeng, Lin Wang, Ling Wang, Hui Zhuang

**Affiliations:** Department of Microbiology, School of Basic Medical Sciences, Peking University Health Science Center, Beijing, China; Virginia Polytechnic Institute and State University, United States of America

## Abstract

This study focused on investigating the pathogenesis seen in specific-pathogen-free (SPF) rabbits following infection with a homologous rabbit HEV isolate (CHN-BJ-rb14) and comparing it to that seen following infection with a heterologous swine genotype 4 HEV isolate (CHN-XJ-SW13). Three of the four animals inoculated with the homologous rabbit HEV became infected, exhibiting an intermittent viremia, obvious fluctuations of liver function biomarkers alanine aminotransferase (ALT) and aspartate aminotransferase (AST), and persistent fecal virus shedding throughout the nine month study. In addition, liver histopathology showed both chronic inflammation and some degree of fibrosis. Both positive and negative-stranded HEV RNA and HEV antigen expression were detected in liver, brain, stomach, duodenum and kidney from the necropsied rabbits. Inflammation of extrahepatic tissue (duodenum and kidney) was also observed. Three of the four rabbits inoculated with the heterologous genotype 4 swine HEV also became infected, showing similar levels of anti-HEV antibody to that generated following infection with the homologous virus isolate. The duration of both viremia and fecal shedding of virus was however shorter following infection with the heterologous virus and there was no significant elevation of liver function biomarkers. These results suggest that rabbit HEV infection may cause more severe hepatitis and prolong the course of the disease, with a possible chronic trend of hepatitis in SPF rabbits.

## Introduction

Hepatitis E virus (HEV), the cause of hepatitis E, appears to be transmitted primarily by the fecal-oral route. In common with Hepatitis A virus with which it shares a number of molecular characteristics, it was initially assumed that HEV only caused acute self-limited disease [1]. However, in the recent years, it has been shown that HEV infection can lead to chronic hepatitis in immune-compromised individuals, such as solid-organ transplant (SOT) patients [2], HIV-positive patients [3] and leukemia patients receiving chemotherapy [4]. The mortality rate associated with HEV infection is generally low (<1%), but it can reach as high as 25–30% in infected pregnant women [5].

HEV is a non-enveloped virus with a positive-sense, single-stranded RNA genome of approximately 7.2 kb, in which the 5′ non-coding region is followed by three partially overlapping open reading frames (ORFs) and a polyadenylated 3′ non-coding region. It is the sole member of the family Hepeviridae [6]. Four genotypes of HEV have been recognized to-date. Genotypes 1 and 2 are restricted to humans, are most commonly found in the developing countries of Asia, Africa and South America [7] and appear to be transmitted mainly via contaminated water. Genotypes 3 and 4 have a more extended host range which includes humans, pigs and other mammals and are responsible for sporadic cases of disease in both developing and industrialized countries either through direct contact with infected animals, or through the consumption of contaminated animal meat and viscera [8].

The first non-human strain of HEV was isolated from a pig in the United States in 1997 and consequently designated swine HEV [9]. The virus has been isolated from various animal species, including chickens, deer, mongooses, foxes, ferrets, rats, bats, wild boars and trout [10]–[18]. In 2009, a new HEV was isolated from farmed rabbits in China [19]. The rabbit HEV strains isolated to-date show 73–77%, 70–76%, 75–82%, 71–77% identity to the genotypes 1, 2, 3, 4 respectively at the nucleotide level and 53–65% identity to avian HEV isolates [20]. Phylogenetically, rabbit HEV isolates are most closely related to genotype 3 [21], [22], although some have suggested that they represent a novel genotype [19], [23]. Under experimental conditions, rabbit HEV has been shown to be able to give cross-species infections in the monkeys and pigs [24], [25]. Swine HEV isolates have also been shown to be able to infect rabbits [26], indicating rabbits may serve as a non-primate small animal model for HEV infection. However, the pathogenesis profile of HEV infection of rabbits has not been clearly defined. Therefore, the aim of this study was to investigate the pathogenesis of rabbit HEV in its natural host and compare it to that given by a genotype 4 swine HEV isolate.

## Materials and Methods

### Ethics Statement

The animal experiments were approved by the Committee of Laboratory Animal Welfare and Ethics, Peking University Health Science Center. This study was performed in strict accordance with the Principles of Laboratory Animal Care (NIH publication no.85Y23, revised 1996).

### Virus Inocula

The rabbit HEV strain CHN-BJ-rb14 (JQ768461) used in this study was recovered from a rabbit fecal sample [24]. The swine HEV strain CHN-XJ-SW13 (GU119961) used was from fecal samples of a pig taken in Xinjiang, China [27]. The fecal samples were diluted in phosphate-buffered saline (PBS; pH 7.4) to make a 10% (wt/vol) suspension, clarified by centrifugation at 5000 rpm at 4°C for 30 min and filtered sequentially through 0.45 µM and 0.22 µM filters. The resulting suspensions were stored at −80°C. The titers of the CHN-BJ-rb14 and CHN-XJ-SW13 inocula used were both adjusted 10^4^ genome equivalents (GE) per milliliter (mL) as determined by semi-quantitative nested reverse transcription PCR (RT-nPCR) [20].

### Animals

3-month old SPF New Zealand white rabbits (2–2.50 kg) were obtained from the Department of Laboratory Animal Science of Peking University Health Science Center. Prior to being infected with HEV, all animals were tested for ALT and AST to establish the baseline and were confirmed negative for anti-HEV antibody by an enzyme-linked immunosorbent assay (ELISA) and negative for HEV RNA in fecal/serum by RT-nPCR.

### Experimental Design

Ten SPF rabbits were divided at random into 3 groups. Group 1 consisted of two rabbits (C1 and C2), which were inoculated intravenously (i.v.) with 1 mL of sterile PBS as negative controls. Group 2 consisted of four rabbits (R1, R2, R3 and R4) and these were inoculated i.v. with 1 m L of the rabbit HEV (strain CHN-BJ-rb14). Group 3 consisted of four rabbits (S1, S2, S3 and S4) which were inoculated i.v. initially with 1 mL of the genotype 4 swine HEV (strain CHN-XJ-SW13). Following their recovery from this initial infection they were inoculated i.v. at 25 wpi with 1 mL the rabbit HEV (strain CHN-BJ-rb14). Each animal was housed in a separate cage and allowed access to food and drinking water *ad libitum*.

### Sample Collection and Processing

Serum and fecal samples were collected at weekly intervals post-inoculation (wpi) and stored at −80°C. Serum samples were tested for levels of ALT, AST and anti-HEV antibody using standard methods [24]. Serum and fecal samples were tested weekly for HEV RNA by nRT-PCR [24]. In addition, bile and a number of different types of tissue and organs including liver, brain, stomach, duodenum, kidney, lung, bladder, and spleen were collected either from animals that died as a result of accidents during the course of the study or those euthanized. One rabbit R1 died as a result of accidents during the course of the study and nine rabbits (C1-C2, R2-R4, S1-S4) survived until euthanasia. Approximately 100 mg of the tissue or organs collected was homogenized in 1 mL of TRIzol reagent (Invitrogen, Burlington, ON, Canada) and clarified by centrifugation at 12,000 rpm for 15 min at 4°C. The supernatants from this centrifugation were harvested and stored at −80°C for later by nRT-PCR. The tissue and organs samples were also processed for histopathology and immunohistochemistry by first being fixed in 10% neutral buffered formalin immediately following sampling. To prevent cross-contamination during necropsy, individually wrapped, sterile disposable materials and new sterile scalpel blades were used for each sample. The organs containing excreta (feces and bile), that can harbor high titres of HEV were sampled last to reduce the potential for cross- contamination.

### Histopathology and Immunohistochemistry

Tissue and organ samples were fixed in 10% neutral buffered formalin, embedded in paraffin and cut into 5 µM serial sections. Slides were stained with hematoxylin and eosin (H&E) and Masson’s Trichrome. The samples were photographed and analyzed using a microscope (Olympus CX31, Japan) equipped with a digital camera with the assistance of Motic Images Plus 2.0 software.

For immunohistochemistry, tissue and organs sections were deparaffinised, hydrated, water-bath heated for antigen retrieval and blocked with the addition of 3% hydrogen peroxide. Sections were then incubated overnight with a 1∶20 dilution of monoclonal antibody (mouse-anti-genotype 1 HEV’S ORF2 antibody, Wantai, Biopharmaceutical, Beijing, China), washed with PBS and stained for visualization using the 2-step plus Poly-HRP Anti-Mouse/Rabbits IgG Detection System (PV-9000, K123307E, ZSGB) combined with DAB substrate (ZLI-9018, K125905A, ZSGB), according to the supplier’s instructions.

### Determination of ALT and AST Concentrations

All rabbits were monitored weekly for 36 weeks following virus challenge. ALT and AST concentrations in serum were measured on the day of collection by using standard methods on a SmartSpec 3000 spectrophotometer (BioRad, CA) [24]. Animals were considered to be suffering from hepatitis when the serum ALT concentration exceeded the pre-challenge ALT level by more than two-fold [28].

### ELISA for Anti-HEV Antibody

All serum samples were tested for anti-HEV antibody using a commercial HEV specific sandwich ELISA assay, based on the E2 protein of genotype 1 HEV (amino acids 394–606 of HEV ORF2), according to the manufacturer’s instructions (Wantai, Biopharmaceutical, Beijing, China). Sample/cutoff (S/CO) values were calculated and values >1 were considered positive [29].

### Detection and Analysis of Positive/Negative-stranded HEV RNA

HEV RNA was extracted from 100 µL of serum/fecal samples or from 500 µL supernatants of tissue homogenates using TRIzol reagent (Invitrogen, Burlington, ON, Canada) according to the instructions. RT-nPCR for detecting positive/negative-stranded HEV RNA was carried out as previously described [24]. Two sets of specific external and internal primer pairs for partial fragments of ORF1 (129–373 nt) and ORF2 (5,983–6,349 nt) were used [24]. Negative and positive controls were included in all assays. RNA extraction and other pre-PCR amplification steps were performed in a separate clean room to avoid cross-contamination. The final amplicons were analyzed by 1% (w/v) agarose gel electrophoresis and visualized by ethidium bromide staining [24]. Selected amplicons of positive/negative-stranded HEV RNA were sequenced to confirm the specific virus inocula used for animal infections.

## Results

### Profile of HEV Infection in the Rabbit

The profile of HEV infection in the rabbit was monitored through the assay of two liver specific enzymes, ALT and AST; through the detection of HEV RNA in feces and by following the level of anti-HEV antibodies in sera. Prior to virus inoculation, all rabbits were negative for anti-HEV antibody and HEV RNA in serum/fecal samples, and had normal levels of ALT and AST (data not shown).

**Figure 1:**
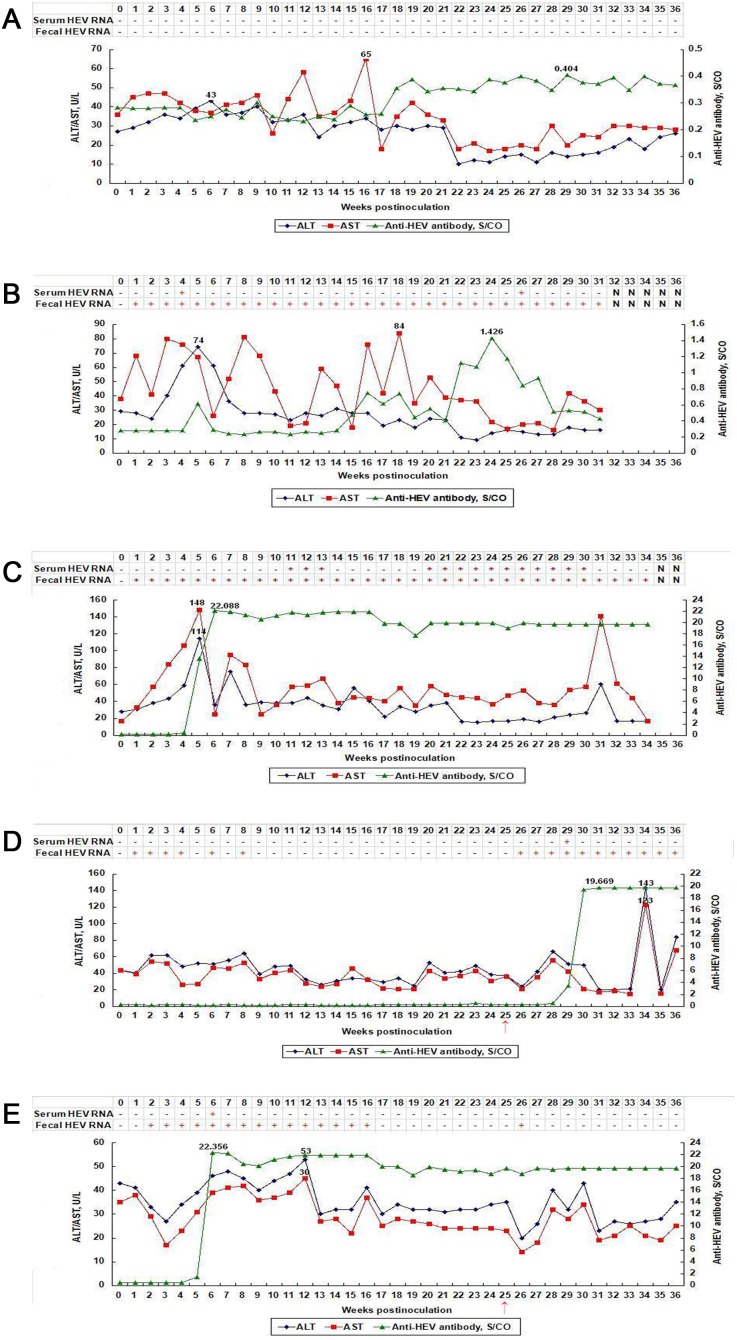
Dynamic seroconversion of anti-HEV, HEV RNA,ALT and AST observed in rabbits. (**A**) Rabbit C1 in group 1 inoculated with sterile PBS. (**B**) Rabbit R1 in group 2 inoculated with rabbit HEV strain. (**C**) Rabbit R3 in group 2 1inoculated with rabbit HEV strain. (**D**) Rabbit S2 in group 3 inoculated with a genotype 4 swine HEV at 0wpi, and rabbit HEV at 25wpi. (**E**) Rabbit S4 in group 3 inoculated with a genotype 4 swine HEV at 0wpi, and rabbit HEV at 25wpi. Note: ↑ indicates group 3 rabbits were inoculated with rabbit HEV strain at 25wpi (when recovered from initial infection).

The control rabbits C1 and C2 were negative for anti-HEV antibody, fecal/serum HEV RNA and had normal ALT and AST levels throughout the study (Fig. 1A, Table 1).

**Table 1:** Detection of HEV RNA in fecal/serum samples collected weekly from rabbits.

Group	Rabbit ID	Positive (+) or Negative (-) in Fecal/Serum Samples at Indicated Wpi																																					
		0	1	2	3	4	5	6	7	8	9	10	11	12	13	14	15	16	17	18	19	20	21	22	23	24	25	26	27	28	29	30	31	32	33	34	35	36	
1^a^	C1	−/−	−/−	−/−	−/−	−/−	−/−	−/−	−/−	−/−	−/−	−/−	−/−	−/−	−/−	−/−	−/−	−/−	−/−	−/−	−/−	−/−	−/−	−/−	−/−	−/−	−/−	−/−	−/−	−/−	−/−	−/−	−/−	−/−	−/−	−/−	−/−	−/−	
	C2	−/−	−/−	−/−	−/−	−/−	−/−	−/−	−/−	−/−	−/−	−/−	−/−	−/−	−/−	−/−	−/−	−/−	−/−	−/−	−/−	−/−	−/−	−/−	−/−	−/−	−/−	−/−	−/−	−/−	−/−	−/−	−/−	−/−	−/−	−/−	−/−	−/−	
2^b^	R1	−/−	+/−	+/−	+/−	+/+	+/−	+/−	+/−	+/−	+/−	+/−	+/−	+/−	+/−	+/−	+/−	+/−	+/−	+/−	+/−	−/−	−/−	+/−	+/−	+/−	+/−	+/+	−/−	+/−	+/−	+/−	+/−	N/N	N/N	N/N	N/N	N/N	
	R2	−/−	+/−	+/−	+/−	+/−	+/−	+/−	+/+	+/−	+/−	+/−	+/−	+/−	+/−	+/+	+/−	+/−	+/−	+/−	+/−	+/−	+/−	+/−	+/−	+/−	+/−	+/−	+/−	+/+	+/−	+/+	+/−	+/+	+/+	+/−	+/−	N/N	
	R3	−/−	+/−	+/−	+/−	+/−	+/−	+/−	+/−	+/−	+/−	+/−	+/+	+/+	+/+	+/−	+/−	+/−	+/−	+/−	+/−	+/+	+/+	+/+	+/+	+/+	+/+	+/+	+/+	+/+	+/+	+/+	+/−	+/−	+/−	+/−	N/N	N/N	
	R4	−/−	+/−	+/+	+/−	+/−	+/−	+/−	+/−	+/−	+/−	+/−	+/−	+/−	+/−	+/−	+/−	−/−	+/−	−/−	+/−	−/−	+/−	+/−	+/−	+/−	+/−	+/−	+/−	+/−	+/−	+/−	+/−	+/+	+/+	+/−	+/−	+/−	
3^c^	S1	−/−	−/−	+/−	+/−	+/−	+/−	+/−	+/−	+/−	+/−	+/−	+/−	−/−	−/−	+/−	+/−	−/−	+/−	+/−	−/−	−/−	−/−	−/−	−/−	−/−	−/−	−/−	+/−	−/−	−/−	−/−	−/−	−/−	−/−	−/−	−/−	−/−	
	S2	−/−	+/−	−/−	+/−	−/−	+/−	−/−	+/−	+/−	−/−	−/−	−/−	−/−	−/−	−/−	−/−	−/−	−/−	−/−	−/−	−/−	−/−	−/−	−/−	−/−	−/−	+/−	+/−	+/−	+/+	+/−	+/−	+/−	+/−	+/−	+/−	+/−	
	S3	−/−	+/+	+/−	+/−	+/−	−/−	+/−	−/−	+/−	+/−	−/−	+/−	+/−	+/−	+/−	+/−	−/−	+/−	−/−	+/−	−/−	−/−	−/−	−/−	−/−	−/−	+/−	+/−	−/−	−/−	−/−	−/−	−/−	−/−	−/−	−/−	−/−	
	S4	−/−	−/−	+/−	+/−	+/−	+/−	+/+	+/−	+/−	+/−	+/−	+/−	−/−	+/−	+/−	−/−	+/−	−/−	−/−	−/−	−/−	−/−	−/−	−/−	−/−	−/−	+/−	−/−	−/−	−/−	−/−	−/−	−/−	−/−	−/−	−/−	−/−	

a Inoculated with sterile PBS (negative controls).

b Inoculated with a rabbit HEV strain CHN-BJ-rb14(JQ768461) (experimental group).

c Inoculated with a genotype 4 swine HEV strain CHN-XJ-SW13(GU119961) at 0wpi and at 25wpi (when recovered from initial infection) inoculated with a rabbit HEV strain CHN-BJ-rb14(JQ768461) (experimental group).

***N*** No samples were collected due to accidental death or being necropsied.

However, an interesting and unexpected result was observed in the rabbits in group 2 inoculated with rabbit HEV. First, persistent fecal shedding of HEV RNA was detected in all rabbits of group 2 from 1 wpi till 36 wpi or to the end of this study (lasting for 9 months). In this group, the peak levels of ALT were at least 3–4 fold higher than baseline values, and peak levels of AST were elevated by approximately 2-fold above baseline levels. Focusing on one of the animals (R3) in this group, it seroconverted for anti-HEV antibodies at 5 wpi and the S/CO values jumped to a peak value of 22 at 6 wpi and maintained at 18–20 level thereafter. In this animal the peak levels of ALT reached was 114 U/L at 5wpi, and a dramatic elevation of AST (148 U/L at 5 wpi) to more than 8-fold above the baseline value of 17 U/L was also seen. Although fecal shedding of virus was detected in R3 throughout the post infection period, viremia was only evident during 11–13 wpi and 20–30 wpi (Fig. 1C, Table 1). The other animals in this group showed a similar disease profile to R3, with the exception that positive sero-conversion was only detected at 22–25 wpi for R1 and the S/CO values were only weakly positive, ranging from 1.072–1.426 (Fig. 1B, Table 1).

In contrast to group 2, the animals in group 3 showed no obvious elevations for ALT and AST following inoculation with inoculated with swine HEV. Rabbit S4 in this group seroconverted for anti-HEV antibodies at 5 wpi, with the S/CO values of anti-HEV jumping to a peak value of 22 at 6 wpi and being maintained at 18–20 thereafter (Fig. 1E, Table 1). Persistent fecal HEV RNA excretion was seen for animal S4 from 2 wpi to 16 wpi, but was not detected thereafter until 25 wpi. All the rabbits in this group were inoculated with the rabbit HEV at 25 wpi. Following this second virus challenge, fecal HEV RNA was detected only once at 26 wpi (Fig. 1E, Table 1). The other animals in this group had a similar disease profile with the exception of rabbit S2, which remained seronegative for anti-HEV antibody until 4 weeks after the second virus inoculation with rabbit HEV at 25 wpi. Following seroconversion of this animal, S/CO values peaked at 20 and were maintained at this level for the 6 weeks until the end of the study. (Fig. 1D, Table 1).

### Liver Histopathology and Immunohistochemistry (IHC)

**Figure 2:**
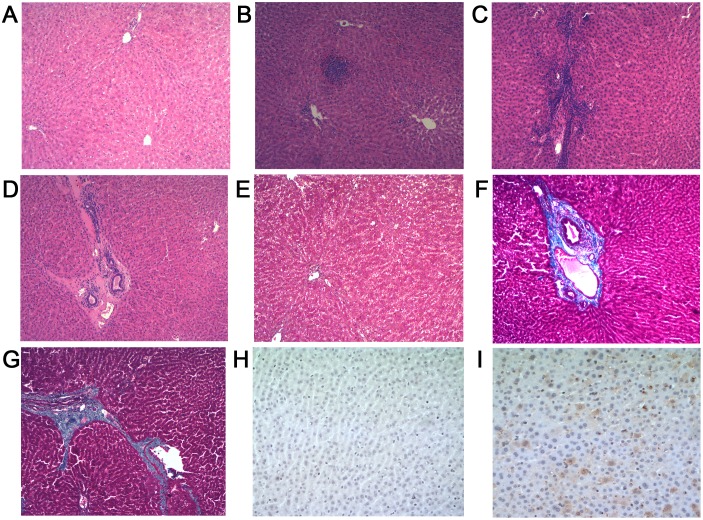
Liver histology. A–D (H & E stain, original magnification, ×10), E–G (Masson’s trichrome stain, original magnification, ×10), H–I (Immunohistochemistry, original magnification, ×40). (**A**) Liver section from a control rabbit with no visible pathological signs of HEV infection. (**B**)-(**C**) Lymphocytes distributed focal or scattered in hepatic lobule, the inflammatory cells gathered along blood vessel walls. (**D**) Chronic inflammatory cells infiltrate the portal area, blood vessel walls thickening associated with fibrosis, local hyaline degeneration. (**E**) No histopathological changes with minimal staining limited to areas immediately adjacent to portal structures. (**F**) Artery wall thickening associated with moderate to severe fibrosis. (**G**) More advanced portal and periportal fibrosis with short fibrous septa. (**H**) Negative immunohistochemistry result for HEV antigen in liver sections from the control rabbits. (**I**) Positive results for HEV antigen in liver sections of experimental groups.

No gross histopathology lesions, no histopathological chronic inflammation or HEV antigens of were observed in liver sections of control group (Group 1) animals (Fig. 2A, 2E and 2H). Histopathology done on animals from Group 2 also showed no gross histopathological lesions, but chronic inflammatory cell infiltrations were observed (Fig. 2B and 2C) and portal fibrosis was obvious (Fig. 2D, 2F and 2G). In addition, HEV antigens were observed in the liver sections of all animals from groups 2 and 3 (Fig. 2I).

### HEV Replication in Extrahepatic Tissue of Infected Rabbits

**Figure 3:**
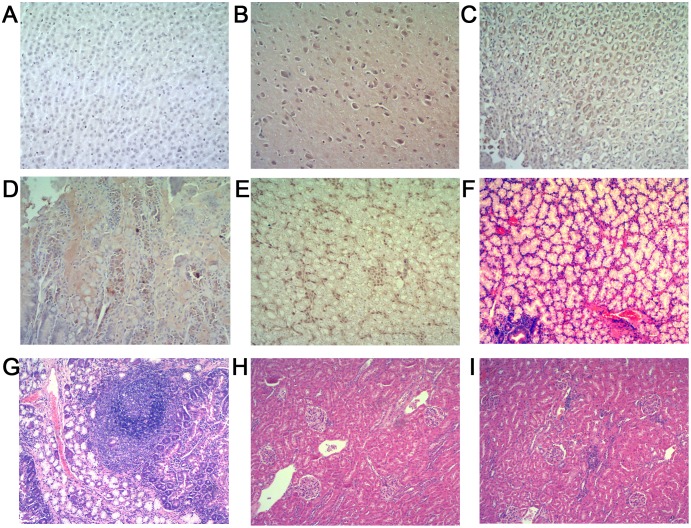
Extrahepatic tissue histology. A–E (Immunohistochemistry, original magnification, ×40), F–J (H & E stain, original magnification, ×10). (**A**) Negative immunohistochemistry result for HEV antigen in extrahepatic tissue sections from the control rabbits. (**B**)–(**E**) Positive results for HEV antigen in brain, stomach, duodenum and kidney. (**F**) Duodenum section from a control rabbit with no visible pathological signs of inflammation. (**G**) A large number of lymphocytes infiltrate mucosal interstitial, focal lymph follicles formed in duodenum sections. (**H**) Kidney section from a control rabbit with no visible pathological signs of HEV infection. (**I**) Multifocal lymphocytes and mononuclear cells infiltrate in renal interstitial.

Positive and negative strand HEV RNA, indicative of active virus replication, was detected in a range of extrahepatic tissues (brain, stomach, duodenum, kidney, bile, lung and bladder) taken from Group 2 infected animals. The positive staining for HEV antigens seen on carrying out IHC staining of brain, stomach, duodenum and kidney sections made from Group 2 infected animals served to provide confirmatory evidence of extrahepatic virus replication (Fig. 3B, 3C, 3D and 3E). Extrahepatic tissue sections made from control group (Group 1) animals were as expected negative for HEV antigen expression (Fig. 3A, 3F and 3H). Interestingly, histological staining of both duodenal and kidney tissues from HEV infected animals (Group 2) showed clear evidence of infiltration with inflammatory cells (Fig. 3G and 3I).

## Discussion

HEV is a well-known pathogen classically associated with acute viral hepatitis [1]. However, a number of more recent studies have shown that infection with genotype 3 HEV can lead to the development of chronic hepatitis in immunocompromised patients [2]–[4]. HEV infection has also been linked to a number of extra-hepatic conditions, including involvement in neurologically based symptomologies [30]. This association of HEV with a range of disease pathologies [31]–[33], coupled to a clear need to establish a tractable small animal model for this pathogen of growing medical importance has stimulated studies of HEV pathogenesis. There have been previous studies that suggested rabbits could be a possible small animal model for HEV infection [26], [34], but none has focused on defining the pathogenic profile of the virus. The most interesting result obtained in the current study focusing on this aspect of HEV biology was that infection of the SPF rabbits used with a homologous rabbit HEV isolate lead in some cases to the development of chronic hepatitis and an associated liver fibrosis. Interestingly this chronic disease pathology was not seen when rabbits were infected with the heterologous genotype 4 swine HEV. Chronic HEV infection has been defined previously as the presence of persistently elevated liver enzyme levels and detectable HEV RNA in the serum and/or stool for at least 6 months [35], and in this study, the rabbits inoculated with rabbit HEV conformed to this definition with persistent fecal shedding and elevated liver enzymes being evident for more than six months after infection. The detection of both the positive/negative-stranded HEV and HEV antigen in brain, stomach, duodenum and kidney, together with the histopathologic changes seen in the duodenum and kidney of HEV infected animals, raises the possibility that the replication of virus in extrahepatic tissues may be responsible some of the clinical symptoms that have been reported. Furthermore, the detection of HEV replication in brain tissue via PCR and immunohistochemistry, provides some evidence based explanation of the neurological signs and symptoms that have been associated with HEV infection [36]. It also indicates that clinicians should consider the possibility of HEV infection in patients with neurologic disorders of unknown etiology.

However, the mechanism of this chronic trend caused by rabbit HEV was unclear. The reported chronic HEV infections occurred mostly in immunocompromised patients and were caused by genotype 3 HEV [2]–[4]. Recently, cases of chronic hepatitis E in immunocompetent patients were reported [37], [38]. Whether the acute viral infection developed to chronic course or not mainly depends on both factors of virus and host. For the interprets of the chronicity of rabbit HEV infection, we thought that phylogenetically the rabbit HEV genome is most closely related to that of genotype 3 HEV isolates, which is considered to be related to developing chronic hepatitis [2]–[4], [37], [38]. Moreover, rabbit HEV was characterized by an insertion of 31 aa in X domain of ORF1 (929–959 aa) [20]. Whether the insertions leading to the chronic trend is unclear and need further study. Besides, long period of nine months persistent fecal virus shedding together with high titer anti-HEV antibody for group 2 rabbits during the disease progress was observed. This result was contradicted with previous study that the neutralizing antibody anti-HEV could neutralize the HEV and therefore the virus should be cleared by high titer anti-HEV antibody [39]. A new report stated that the ‘non-enveloped’ virus HEV in blood can derive the host’s membranes, therefore can escape from the neutralizing antibodies of HEV [40]. This may be another factor responsible for the chronicity of HEV infection.

In summary, this study has provided the first experimental evidence that SPF rabbits experimentally infected with a homologous rabbit HEV isolate develop signs of chronic hepatitis. Given the increasing medical importance of HEV infections and their association with chronic symptomology in some patient groups, the underlying mechanisms responsible for the chronic pattern of pathogenesis seen in this study deserves further study.
